# The Tempered Polymerization of Human Neuroserpin

**DOI:** 10.1371/journal.pone.0032444

**Published:** 2012-03-06

**Authors:** Rosina Noto, Maria Grazia Santangelo, Stefano Ricagno, Maria Rosalia Mangione, Matteo Levantino, Margherita Pezzullo, Vincenzo Martorana, Antonio Cupane, Martino Bolognesi, Mauro Manno

**Affiliations:** 1 Institute of Biophysics, National Research Council of Italy, Palermo, Italy; 2 Department of Physics, University of Palermo, Palermo, Italy; 3 Department of Biomolecular Sciences and Biotechnology, Institute of Biophysics CNR and CIMAINA, University of Milano, Milan, Italy; Università di Napoli Federico II, Italy

## Abstract

Neuroserpin, a member of the serpin protein superfamily, is an inhibitor of proteolytic activity that is involved in pathologies such as ischemia, Alzheimer's disease, and Familial Encephalopathy with Neuroserpin Inclusion Bodies (FENIB). The latter belongs to a class of conformational diseases, known as serpinopathies, which are related to the aberrant polymerization of serpin mutants. Neuroserpin is known to polymerize, even in its wild type form, under thermal stress. Here, we study the mechanism of neuroserpin polymerization over a wide range of temperatures by different techniques. Our experiments show how the onset of polymerization is dependent on the formation of an intermediate monomeric conformer, which then associates with a native monomer to yield a dimeric species. After the formation of small polymers, the aggregation proceeds via monomer addition as well as polymer-polymer association. No further secondary mechanism takes place up to very high temperatures, thus resulting in the formation of neuroserpin linear polymeric chains. Most interesting, the overall aggregation is tuned by the co-occurrence of monomer inactivation (i.e. the formation of latent neuroserpin) and by a mechanism of fragmentation. The polymerization kinetics exhibit a unique modulation of the average mass and size of polymers, which might suggest synchronization among the different processes involved. Thus, fragmentation would control and temper the aggregation process, instead of enhancing it, as typically observed (*e.g.*) for amyloid fibrillation.

## Introduction

Serpins (SERine Protease INhibitor) are highly conserved proteins that control serine protease activities in different extracellular environments [1]. Several mutations identified in humans are at the origin of a large class of pathologies, known as serpinopathies, which share a common molecular mechanism: the polymerization of a mutant serpin and its accumulation within the endoplasmic reticulum (ER) [2]. Such event causes toxicity (gain of function phenotype) in the tissue producing the mutant serpin, or the lack of protease inhibition in the proper extracellular environment (loss of function phenotype). Serpins are composed of three β-sheets (A–C) and an exposed mobile reactive centre loop (RCL), acting as a target for the serine protease [3]. The protease cleaves the RCL triggering the insertion of the RCL into the A β-sheet as a strand; however, the protease remains covalently trapped, almost irreversibly, as an acyl enzyme intermediate [4]. The mechanism of serpin polymerization is intimately related to their inhibitory function [5], [6]. Indeed, unlike other globular proteins, native serpins are folded in a metastable conformation, and reach a more stable state by interlocking the RCL into the A β-sheet, thus reducing its mobility [7]. This is achieved upon RCL cleavage, or by adopting a distorted conformation in which the RCL is fully or partially inserted into the A β-sheet without cleavage (latent conformation) [8]. Alternatively, the RCL may be inserted into the sheet of a neighbor serpin molecule thus forming dimers and eventually polymers [9]. For these characteristics, serpin polymers are ordered structures, which do not require extensive protein unfolding and, in general, do not activate the unfolded protein response or other stress signaling pathways from the ER [10]. This marks the peculiarity of serpinopathies within the larger class of protein conformational diseases, which include important disorders such as amyloidoses and other neurodegenerative diseases [11].

Here, we study the mechanism of polymerization of human neuroserpin (NS), a secretory protein produced mainly in neurons, playing a role in axonogenesis and in regulating synaptic plasticity through the inhibition of tissue-type plasminogen activator (tPA) [6], [12], [13]. It is also associated to Alzheimer's disease through a still not clear role [14], [15]. NS is related to the autosomal dominant dementia Familial Encephalopathy with Neuroserpin Inclusion Bodies (FENIB) [16]. Selected point mutations promote NS polymerization [17]–[19] and its ER retention, with a high genotype-phenotype correlation [20], thus providing a clear example of how serpin polymerization causes human disease [21], [22]. Beyond its clinical relevance, NS is a suitable model for biophysical studies of serpin polymerization due to its close structural homology with the archetypal serpin α1-antitrypsin (AAT) [23], whose deficiency represents the most common serpinopathy [24], but also due to its capability of forming polymers by thermal stress [17], [25], [26] or under acidic conditions [27], even in its wild-type form.

The classical model for serpin polymerization [9], understood for the Z variant of AAT, foresees the formation of an intermediate state in the pathway to polymerization [8], [28], which can accept the insertion of the RCL from a neighbor serpin molecule forming “loop-sheet” polymers [29]. The same mechanism is likely responsible for the formation of loop-sheet polymers of other mutant variants of AAT [30]–[32], and NS [15], [26]. However, an alternative serpin polymerization mechanism has been proposed, based on the crystal structure of a closed dimer of antithrombin grown under acidic conditions. In this model, polymers would form by iterative domain swapping of a hairpin composed by strands s4A and s5A [33]. A more recent crystallographic study revealed alternative domain swapping involved in the structure of an antitrypsin trimer [34]. Although the relevance of this model for the polymerization in vivo is not clear yet, it suggests that different environmental conditions and specific point mutations may lead to structurally diverse polymers, through polymerization mechanisms that are still a matter of debate. Detailed knowledge of serpin structural behavior (intermediate conformations, conversion to latency, polymerization) is fundamental for the design of therapeutic aids helping prevent diseases associated with serpin polymerization.

NS forms polymeric structures upon heating above physiological temperatures [17]. As shown by our previous work [25], [26], neuroserpin maintains a folded conformation and polymerogenic properties up to high temperatures, even if the molecular structure of polymeric and latent conformers may be slightly different above 80°C. In the present work, we study the mechanism of polymerization of human neuroserpin at different temperatures and concentrations by static and dynamic light scattering (LS) and size exclusion chromatography (SEC), two techniques suitable for monitoring the mass and size evolution of protein aggregates, as well as by intrinsic tryptophan photoluminescence (PL) to reveal the conformational changes occurring during aggregation. Our experiments, in keeping with previous results [15], [26], confirm that the polymerization is rate limited by two processes: activation of an intermediate, and dimerization of such an intermediate with a native monomer. Moreover, our data show that the late stage of aggregation is dominated not only by the process of monomer addition to polymers, but also by polymer-polymer association. Interestingly, we observe a remarkable correlation between the hydrodynamic radius and the mass of polymers, which highlights the supramolecular structure of NS aggregates as polymeric chains formed only by end-to-end association, and not by other secondary mechanisms, such as branching or lateral association. The same processes seem to operate in a large range of temperatures.

The most striking observation emerging from our experiments is a modulation in the growth of the polymer mass and size with time, which implies the occurrence of fragmentation, or any kind of depolymerization such as simple monomer release from polymers, in keeping with analogous observation for AAT polymerization [35]. This behavior is quite unique in the scenario of protein aggregation. For example, in the case of amyloid fibrillation, aggregation is typically enhanced by fragmentation [36]. The analogy with the oscillations in the length of microtubules prompted us to consider some kind of synchronization or feedback among the different processes involved [37]. However, a rationale for such a re-entrant growth can be found qualitatively by assuming the occurrence of depolymerization below a given polymer size, and the concomitant process of latentization that sequesters the active monomers thus tempering the progression of aggregation. The idea of a fragile interlocking of the RCL in the polymeric structures, prompted by our observation of a fragmentation mechanism, finds a counterpart in the lack of stability of the NS-tPA complex. Indeed, contrary to other serpins, NS is a transient inhibitor of tPA and the acyl-enzyme NS-tPA complexes are short-lived, [23], [38]. The fragility of both polymers and complexes may be related to the relative stability of the different neuroserpin conformers, and may be relevant both for the pathology and for the physiological role of NS.

## Results

### Kinetics of polymerization studied by LS

**Figure 1:**
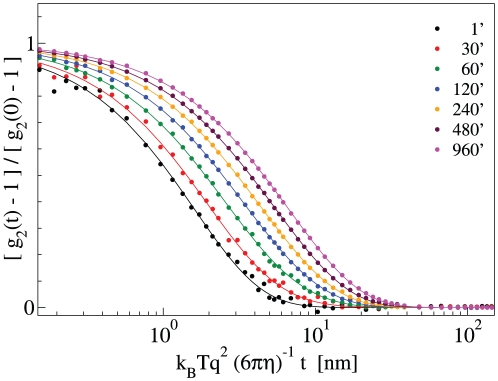
Intensity autocorrelation function *g_2_(t)* of a 8.5 µM NS solution during polymerization at 45°C at selected times, as shown in the legend (coloured circles), and the relative fit by exponential funtions (solid curve). The time axis is renormalized by the following thernodynamic and experimental quantities: *q* is the scattering vector, T the temperature, k_B_ the Boltzmann constant, and η the solvent viscosity.

Simultaneous dynamic and static LS measurements were performed at different temperatures from 37 to 85°C, at concentrations around 4.5 µM (except above 70°C, where the concentration was reduced down to 1.5 µM). Fig. 1 shows typical intensity autocorrelation functions measured at selected times during polymerization at 45°C. The intensity autocorrelation functions in the course of the kinetics were fit by both a regularization scheme and a double exponential function, in order to derive the hydrodynamic radii *R_h_* of monomeric and polymeric species, as reported in the Methods section [39]–[41]. The z-average hydrodynamic radius has been also calculated by the robust cumulant method [42]. All the fitting procedures yield perfectly consistent results. Data in Fig. 1 indicate the increase of the polymeric fraction accompanied by a time dependent increase of the polymer hydrodynamic radius, that reaches a value of *R_h_*∼15 nm after 960 minutes of incubation at 45°C. Upon incubation at high temperature, neuroserpin forms polymers up to a hydrodynamic radius of a few tens of nanometers, in agreement with Transmission Electron Microscopy images (data not shown. See also Fig. S1) [25].

**Figure 2:**
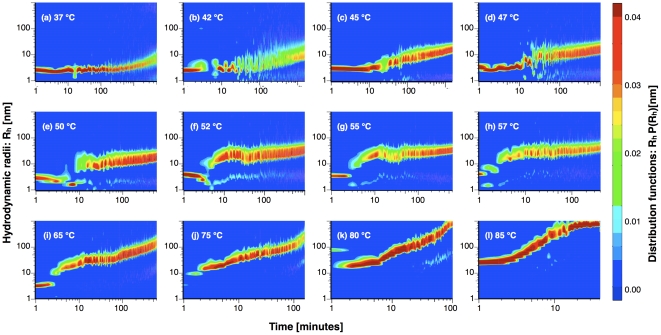
Distribution functions of hydrodynamic radii versus incubation time at different temperatures (as specified in each panel).

Fig. 2 displays the time evolution of the distribution functions *P(R_h_)* of the hydrodynamic radii *R_h_* at different temperatures. The *P(R_h_)* values in the figure were color-coded so that the red color denotes the most frequent *R_h_* of the population. In the intermediate range of temperatures we clearly note that the distribution functions *P(R_h_)* are mainly bimodal, and composed of one monomer peak and one polymer peak. The polymer fraction grows uniformly with a variance essentially proportional to the average. In other words, the distribution of polymer hydrodynamic radii *P(R_h_)* shifts to larger *R_h_* without changing its shape on a logarithmic scale and thus exhibits a self-similar shape in the course of the kinetics.

The kinetics at high temperature deserves specific attention (Figs. 2k, 2l). At 85°C polymerization takes place in a few minutes leading to the formation of micron-sized aggregates, as already reported in our previous work [25]. At later stage, the diffusion of such aggregates is affected by their large-size and entanglement, as observed in jammed polymer networks [43] (Fig. S2). A detailed study of this late stage is beyond the scope of the present work. Here, it is worth to remark that the aggregation in the early stage (i.e. that occurring in the first 10÷30 minutes) is akin to that occuring at lower temperatures and leading to the formation of serpin-like polymers.

### Occurrence of different processes: latentization, monomer addition, fragmentation and polymer-polymer association

It is worth noting that in the polymerization kinetics (Fig. 2) a residual fraction of monomeric species is maintained even after the formation of protein aggregates, as clearly seen up to 57°C. A mechanism that prevents monomer depletion is related to the inactivation of neuroserpin monomers due to the formation of latent conformers (latentization), both at low and high temperatures [15], [25].

**Figure 3:**
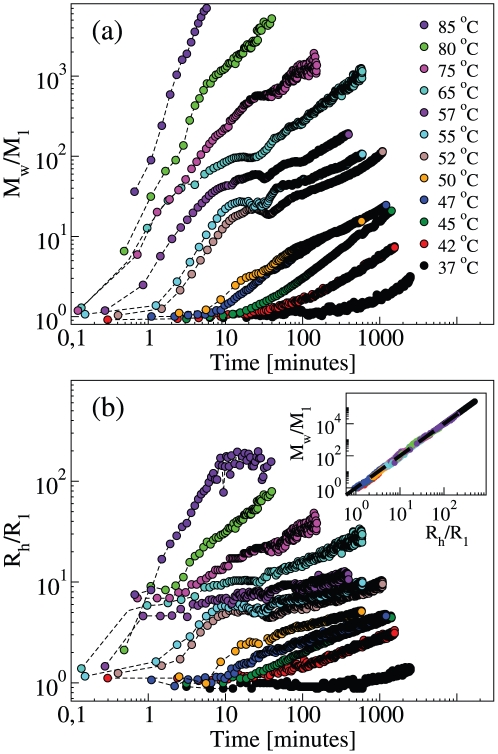
Kinetics of neuroserpin polymerization at different temperatures. (a) Weight average mass *M_w_* normalized by the monomer mass *M_1_*. (b) z-average hydrodynamic radius *R_h_* normalized by the monomer radius *R_1_*. Inset: Correlation between *M_w_* and *R_h_*; the dashed line is a power law with exponent 2.

From the static scattering intensity, one obtains the weight average mass *M_w_* of protein aggregates. Fig. 3a shows the kinetics of *M_w_* at the various temperatures, and Fig. 3b shows the z-averaged hydrodynamic radii in the same conditions. The kinetics of polymerization exhibits two stages: an initial fast formation of small polymeric species, growing in mass and size, is followed by the aggregation of larger polymers with a lower rate. These two stages are not clearly separated at the lower temperatures, while they can be clearly recognized above 50°C.

**Figure 4:**
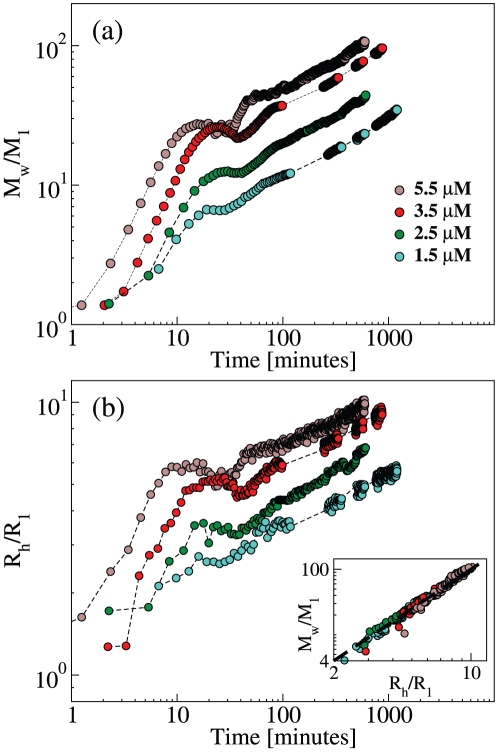
Kinetics of neuroserpin polymerization at 55°C and at different concentrations. (a) Weight average mass *M_w_* normalized by the monomer mass *M_1_*. (b) z-average hydrodynamic radius *R_h_* normalized by the monomer radius *R_1_*. Inset: Correlation between *M_w_* and *R_h_*, the dashed line is a power law with exponent 2.

In the intermediate range of temperatures, we observe a peculiar behavior at the transition between the two stages. The signal related to *M_w_* and *R_h_* is not a monotonous function of time, but follows a modulated, pseudo-oscillatory behavior. At temperatures around 55°C, a reduction of the average mass and size is evident, implying the fragmentation or depolymerization of polymer aggregates (Figs. 2 and 3a,b). The above polymerization feature is observed at 55°C at different protein concentrations, with a concentration-dependent characteristic time (Fig. 4). At 55°C, the quasi-oscillatory behavior and the presence of fragmentation is even more striking than at the other temperatures.

The average mass and hydrodynamic radius continue growing even at the late stage (Figs. 2, 3, 4). In general, if the aggregation by monomer addition were not preceded by nucleation or activation, the polymer mass could grow only to a value which is less or equal to three times the initial value [44]. In Figs. 3 and 4, we may recognize at long times an asymptotic power-law behavior in the growth of polymer mass and size: *M_w_∼t^z^* and *R_h_∼t^z/d^*. By simultaneous inspection of the curves at different temperatures, the best exponent turns out to be *z* = 2/3. This indicates that polymerization proceeds not only by monomer addition but also by polymer-polymer association (or fragmentation). Indeed, if only dimerization and monomer addition took place, the weight average mass would reach a stationary value due to monomer consumption. Chiou et al. assigned the larger rate of association to the dimerization process and considered polymer-polymer association as negligible [15]. Those results are based on single molecule fluorescence experiments, and therefore they are intrinsically sensitive to the low molecular mass species, while our experiments based on LS are sensitive to larger species. Thus, we may consider that the data reported here and by Chiou et al. are not incompatible: they simply reflect a different focus on large and small aggregates, respectively.

### Neuroserpin forms random polymers, with no secondary mechanism

The growths of *M_w_* and *R_h_* are correlated, as shown in the inset of Figs. 3b and 4b. The two quantities are related by a power-law: *M_w_/M_1_ = (R_h_/R_1_)^d^*, where *M_1_* = 46 kDa and *R_1_* = 3 nm are respectively the molecular mass and the hydrodynamic radius of neuroserpin monomers, and the exponent *d = 2±0.1* is the apparent fractal dimension, a parameter related to the packing and self-similarity of aggregates. For instance, the value *d = 2* is typical of random polymers, as expected in the present case [45]. Interestingly, the same type of correlation extends over all kinetics and at all temperatures and concentrations. Therefore, the same type of structure is formed with no secondary aggregation mechanisms, such as lateral association [46], branching [47], or secondary nucleation [48], [49]. It is worth noting that the power-law starts from the monomer size, which may be considered the building unit of polymer structures. In other words, the persistence length of polymers is of the order of the monomer size, in keeping with TEM images (Fig. S1).

### Kinetics of polymerization by SEC

**Figure 5:**
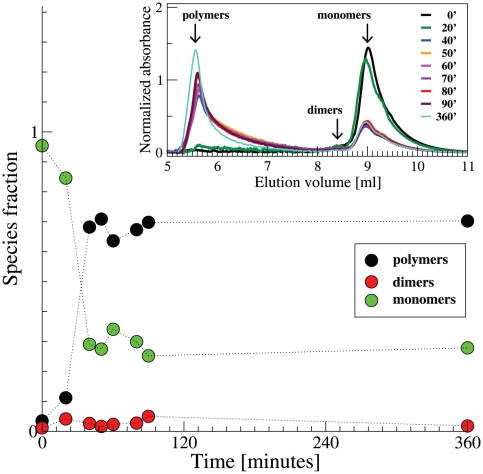
Kinetics of neuroserpin polymerization at 55°C monitored by time-lapse SEC: fraction of monomeric (green circles), dimeric (red circles) and polymeric (black circles) species. Inset: Chromatograms at different times upon incubation at 55°C.

LS measurements are intrinsically more sensitive to molecules and aggregates with a higher molecular mass. In order to confirm the kinetics results reported above with a focus on the monomer and small polymers, we performed time-lapse SEC experiments. The inset of Fig. 5 displays the chromatographic profiles of samples incubated at 55°C. The corresponding percent amounts of monomers, dimers and polymers are reported in Fig. 5. These results confirm the persistence of the monomeric fraction, as obtained in the analysis of LS data. At about one hour of incubation we observe a temporary reduction of the polymeric fraction, which is then restored to higher values. Such a reduction occurs immediately after the initial main increase of the polymeric populations, which coincides with the first stage of the kinetics observed by LS. This is an independent confirmation of the existence of a fragmentation mechanism, which complements the more statistically significant LS experiments. Further, it evidences that the fragmentation includes not only a break of extended polymers but also monomer release from polymers.

### Kinetics of polymerization by tryptophan PL

**Figure 6:**
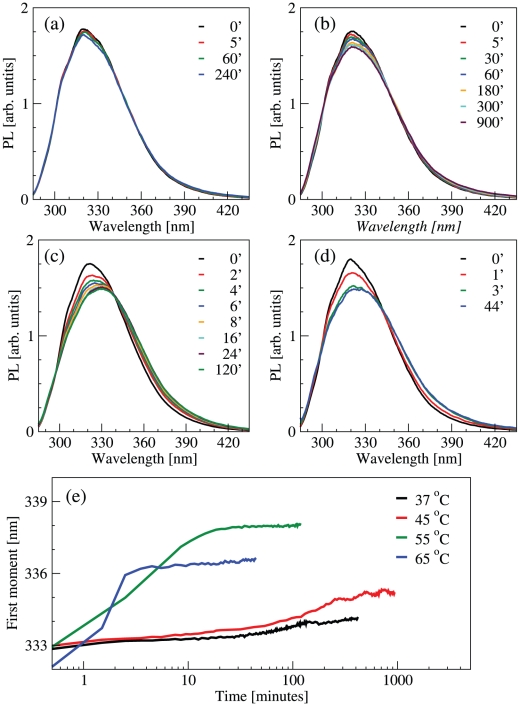
Kinetics of neuroserpin polymerization monitored by time-lapse PL. (a–d) Emission spectra during the kinetics at 37°C, 45°C, 55°C, 65°C. (e) Kinetics of the first moment of the emission bands in the previous panels.

The association of molecules into polymers implies a change in the native conformation. In order to monitor such a change, we measured PL spectra during the kinetics at 37, 45, 55, and 65°C (Figs. 6a, b, c, d). During polymerization the tryptophan luminescence band exhibits a progressive red shift. Accordingly, the signal intensity decreases, probably due to the thermal activation of non-radiative channels, which quench the luminescence from the excited electronic state. Fig. 6e shows the results of the first moment analysis on the steady-state PL of neuroserpin tryptophans. In general, a structureless red-shifted band in tryptophan PL points to a polar environment [50]; for example, a red-shift is observed in protein thermal unfolding [51]. Neuroserpin has three tryptophans, at positions 279, 190 and 154. The latter is on the F helix, which is likely to play a relevant role in the polymerization mechanism and to move in order to leave space for the RLC insertion into β-sheet A [52]. Thus, the formation of an active intermediate and the subsequent association may be reasonably related to a more solvent exposed Trp154 residue. Here, we observe that the shift is complete within the first stage of kinetics, that is while the process of activation and dimer formation is prevalent. No large conformational changes occur at a later stage, which is therefore dominated by the association of already formed polymers.

## Discussion

### “Universal” features of NS polymerization

**Figure 7:**
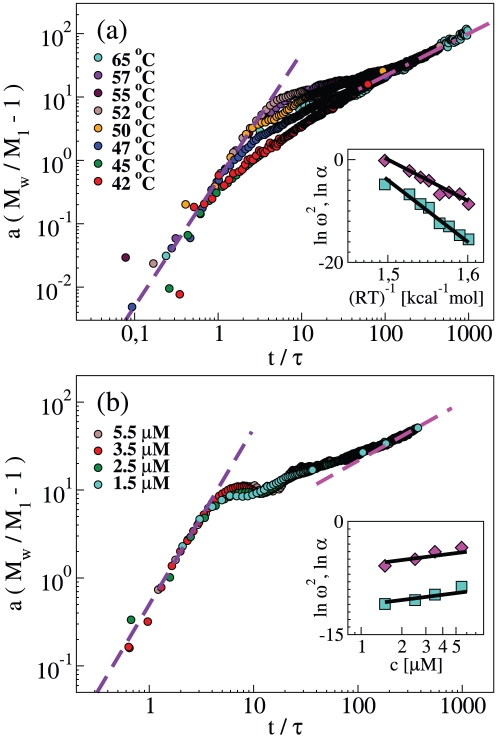
Kinetics of neuroserpin polymerization at different temperatures (panel a) and concentrations (panel b). Weight average mass from figs. 3a and 4a, respectively, rescaled as explained in the main text. Insets: Kinetics rates obtained from the scaling parameters *ω^2^* (squares) and *α* (diamonds).

Even if the polymerization kinetics exhibits a certain level of complexity, one may estimate the initial rate of polymer growth, as well as the final association rate, and gain some insight on the general physical mechanisms underlying the reported NS polymerization kinetics. To this purpose, a double phenomenological scaling of LS data was performed to match both the initial and the long-time asymptotic part of the kinetics at each temperature and concentration. The time axis was rescaled by using a characteristic time *τ*. An effective amplitude parameter *a* was used to rescale the polymer mass in excess with respect to monomer, that is the quantity *M_w_/M_1_−1*. The resulting plot is reported in Fig. 7 and shows that the initial and final stages of the measured polymerization kinetics obey some general (“universal”) behavior.

The curve that best describes the initial mass growth is a quadratic law, with the form: *M_w_/M_1_−1 = ½ ω^2^t^2^*, where the rate *ω = [1/(aτ^2^)]^1/2^*, related to the scaling parameters, depends upon temperature and concentration. The latter expression may be considered as an expansion at time zero of *M_w_/M_1_*, where the first time derivative is zero and the second one is *ω^2^*. In the classical model by Oosawa and coworkers [53] and in the subsequent extensions [54], the initial growth of the aggregate concentration with a power-law with an exponent *n+1* implies that the aggregation is initiated by the formation of a nucleus of *n* monomers. Roberts and collaborators have extended this scheme by including a reversible conformational transition preceding nucleation [55]. In the present case, the initial quadratic growth of data in Fig. 7 implies that the initial polymerization stage is dominated by the sequence of two processes: a monomer conformational transition (the activation process) with a rate *κ_A_* and a dimerization process between a native and an activated monomer with a concentration-dependent rate *c·κ_D_*, where *c* is the molar concentration. Indeed, if the initial growth were due to the association of two native monomers, the weight averaged mass would have a non-null first-derivative at time zero, and hence a linear growth [41], [54]. On the other hand, if two activated monomers were required to initiate polymerization, a higher power-law behavior (namely *t^3^*) would have been observed. Therefore, the quadratic behavior of the onset of aggregation is a further endorsement of the dimerization between a native and an activated monomer [15]. The set of equations, which describes the processes involved in polymerization, along with the expansion at time zero, is reported as Supporting Information (Tables S1, S2).

The second time-derivative of *M_w_/M_1_* is given by the product of the rates of two processes *ω^2^ = 2κ_A_κ_D_c*. The linear concentration dependence of the parameter ω^2^ at 55°C is estimated in the inset of Fig. 7b. One obtains: κ_A_κ_D_ = 8 s^−2^ M^−1^. The parameter ω has been calculated at different temperatures, and it is shown in an Arrhenius plot in the inset of Fig. 7a. This indicates that the same processes operate in this range of temperatures. One obtains an enthalpy change for the activation and dimerization of 120 kcal mol^−1^. If we take this result and extrapolate at 45°C we obtain a value κ_A_κ_D_ = 0.02 s^−2^ M^−1^, which is one order of magnitude lower than the value estimated by Chiou et al. [15]. Again, this discrepancy can be rationalized by considering the different sensitivity to polymer size, which may bias both their data and our data in opposite directions.

Beyond the growth at low incubation times, we may rationalize also the behavior at very long incubation times. Indeed, the average size of polymers has a limited and constant polydispersity (Fig. 2), and the curves of the weight average mass tend asymptotically to a power-law. In Fig. 7, the second phenomenological scaling was performed in order to report the asymptotic behavior of each curve to the power-law *t^2/3^*. With these features, we may argue that the association rate has a mass dependence, which is a homogeneous function of mass with an exponent *λ* [56]. Such condition typically occurs when the aggregation mechanism is limited by diffusion [57], so that *λ* is the reciprocal of the scaling exponent between aggregate mass and hydrodynamic radius: *λ = −d^−1^* [44]. In such a case the analytical solution for the weight average mass is: *M_w_/M_1_∼(αt)^z^*, where *z^−1^ = 1−λ*. In the present case, we find *d* = 2 (insets of Fig. 3b and 4b), and *z* = 2/3 (Fig. 7) that consistently imply *λ* = −0.5. The same type of growth would be found if the fragmentation kernel were also a homogeneous function. In such a case the structure of the kernel is less straightforwardly related to aggregate properties [58]. However, the same type of power-law may be found asymptotically under given conditions [59]. In order to obtain a not stationary solution, as in the present case, the breakup rate at large cluster size should be overwhelmed by the rate of polymer-polymer association.

The rate *α* may be estimated by the scaling parameters of Fig. 7: *α = ωa^−1^*. Its concentration dependence at 55°C is shown in the inset of Fig. 7b. One obtains *α/c* = 3000 s^−1^ M^−1^. The same parameter, obtained at different temperatures, can be drawn in an Arrhenius plot as in the inset of Fig. 7a. The enthalpy change related to this parameter, derived from the slope of the Arrhenius plot, is 80 kcal mol^−1^. By extrapolating this result to 45°C we obtain a value *α* = 55 s^−1^, which is close to the value obtained by Chiou et al. for a rate of association [15]. Indeed, the parameter α represents a generic affinity rate, since it is not only related to the association mechanism but also in a hidden way to the fragmentation. Although its physical meaning is not clearly worked out, it is clearly related to the energy of activation of all the processes prevalent at the late stage of aggregation and mainly to that of polymer-polymer association.

### Kinetics of different processes: a clue from basic models

Our experiments allow extending the kinetic scheme of neuroserpin polymerization, by taking into account the different processes involved. The early stages of aggregation are rate-limited by two processes: (i) the activation of monomers, with a rate *κ_A_*, through a conformational change that allows them to initiate the polymerization and (ii) the formation of dimers, with a rate *κ_D_*, by the association of a native with an activated monomer. The elongation of polymeric structure proceeds beyond dimerization by two other processes: (iii) the addition of monomers to already formed polymers, with a rate *κ_M_* and (iv) the association of polymers of different lengths, with a rate *κ_P_*. The latter process has been first clearly observed in the present work for NS aggregation. The main new qualitative feature added by the results here reported is represented by the occurrence of a modulation, and sometimes a decrease, of the polymer mass and size. Such consideration implies the introduction of an additional process, (v) polymer fragmentation, with a rate *κ_F_*. In the case of neuroserpin we know that a further process takes place, i.e. (vi) the formation of latent monomers, with a rate *κ_L_*.

A general kinetic scheme accounting for all the above processes can be built to produce numerical profiles of experimental available quantities (e.g. scattered intensity). As a matter of fact, only a few model parameters can be set on the basis of our experimental results, and more details should be known to aim at a quantitative fitting of the data. However, a lot can be learned from a basic model, which is able to spot qualitatively the general features of the polymerization kinetics. In the following we sketch the role of the different processes involved by calculating the growth of the weight average mass for given values of the rates. The complete kinetic scheme of equations used is reported as Supporting Information (Table S2). In such an example, we take equal value for the rates of the three processes of activation, dimerization and monomer addition (*κ_A_ = κ_D_ = κ_M_ = 1*).

**Figure 8:**
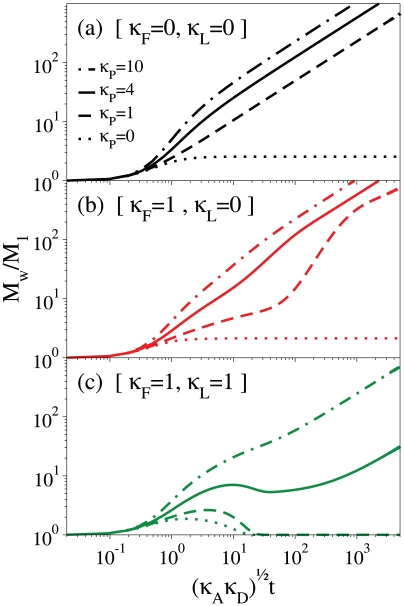
Polymerization kinetics due to different processes. The y-axis displays the weight average mass, the x-axis displays the time normalized by using the rates of activation (*κ_A_*) and dimerization (*κ_D_*). (a) Kinetics at different rates of polymer-polymer association (*κ_P_*). (b) Kinetics as in the previous panel by addition of a fragmentation process from polymers below 31 units. (c) Kinetics as in the previous panel by addition of a latentization process from activated monomers.

In Fig. 8a, we show qualitatively the effects of including polymer-polymer association in the overall aggregation process. The kinetics of weight average mass *M_w_* are plotted for different values of the rate *κ_P_* of polymer-polymer association. As recalled above, in the absence of polymer-polymer association (*κ_P_ = 0*, dotted curve in Fig. 8a) *M_w_* reaches a plateau due to monomer depletion. The increase of the association rate *κ_P_* causes *M_w_* to increase. In this example the increase is linear as a function of time, since we used for simplicity a constant size-independent rate. A more realistic representation would require a size-dependent rate, as discussed above, yielding an asymptotic power-law with a sub–linear growth.

The other process required by our experiments is polymer fragmentation. Indeed, monomer release from polymers is suggested by our experiments (Fig. 5), while it is not clear yet whether other types of polymer breakdown are occurring. From the observation of the small polydispersity in the distribution of hydrodynamic radii (Fig. 2), we may argue that the fragmentation rate should be a smooth function of polymer size, if not a homogeneous function (as discussed above). Another possibility would be that fragmentation is restricted to a given size of polymers. This possibility is particularly sound since it naturally introduces a separation of the time-scales of fragmentation and polymer-polymer association even if they share similar rates. In Fig. 8b, we show the kinetics of *M_w_* at different rates of polymer-polymer association calculated by including the release of monomers from polymers of size below 31 units, and with a rate *κ_F_ = κ_M_ = 1*. While in the absence of polymer-polymer association M_w_ reaches a stationary value (*κ_P_ = 0*, dotted curve in Fig. 8b), the plateau is lost due to the prevalence of association at a later stage, and *M_w_* grows with a sizeable modulation of the slope, especially for smaller values of *κ_P_*.

Last, we consider the effect of the other process peculiar to serpin polymerization, which is the latentization. We calculated the kinetics of *M_w_* by including latentization from the active monomers, as suggested in ref. [15], with a rate *κ_L_ = κ_M_ = 1*. The results for different rates of polymer-polymer association are shown in the curves of Fig. 8c. We note that when such association is suppressed (*κ_P_ = 0*) the latent monomer acts as a sink with respect to the other species, since latentization was assumed to be irreversible. For given values of the association rates (*κ_P_ = 4* in the example shown in Fig. 8), the co-occurrence of fragmentation and latentization determines a transient reduction of the weight average mass, which resembles qualitatively our experimental findings. Indeed, after the activated formation of dimers and small polymers, fragmentation preserves the amount of monomeric species that are then sequestered by latentization. At later time polymer-polymer association becomes overwhelming and sustain a monotonous growth of the average mass.

**Figure 9:**
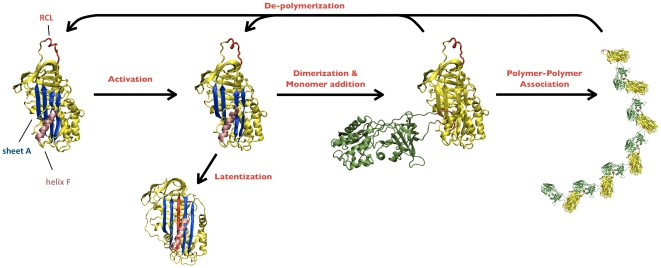
Scheme of neuroserpin polymerization. The arrows and the text highlight the different processes involved. The small bars on the structure of the native protein (left) point to the main structural features of neuroserpin discussed for the polymerization process: the reactive center loop (RCL), the β-sheet A and the α-helix F.

This scheme, pictorially described in Fig. 9, is able to capture the main qualitative features of our experimental data, as shown by the continuous curve in Fig. 8c. However, it is still not complete to fit quantitatively the overall kinetics. We are not aware of any alternative models explaining the details of such a unique aggregation process.

It is of some interest to compare our observations of NS polymerization with analogous features reported for different aggregating macromolecular systems. In particular, the phenomenon of aggregate fragmentation is faced in the literature of amyloid fibrils. In such a field, the effect of fragmentation in general is linked to an increase of amyloid toxicity and enhancement of fibrillation due to the fact that more active ends become available for elongation [36], [47].

The modulated increase of neuroserpin polymer mass is reminiscent of another physiologically important case, which is the growth and oscillation of microtubules [37]. The growth of microtubules is controlled by ATP hydrolysis, via a cycle of processes: (i) active ATP-tubulins form active ATP-polymers, (ii) active ATP-polymers turn into inactive ADP-polymers, (iii) the latter undergo depolymerization and release ADP-tubulin, (iv) ADP-tubulin is activated to ATP-tubulin to restart the cycle [60]. The synchronization of these processes, under particular protein and ATP concentrations, generates sustained oscillations in microtubule length. In the present case, we are only facing a modulation of the signal and a pseudo-oscillatory behavior at the crossover between two kinetic stages. Note also that the available models for microtubule oscillation succeed in reproducing experimental data only by introducing some kind of concentration-dependent rate in one of the processes involved [60]–[62]. On the contrary, based on what has been reported here, we may conclude that the basic model of polymerization, based on the first order processes of activation, latentization and fragmentation and the second order processes of monomer and polymer association, is not sufficient and some other “ingredient” is missing. We have shown that an interesting hint comes from an ad hoc kernel built to allow the fragmentation only of polymers of a specific size. Further advancements may take into account more complex phenomena. However, this would require more detailed experimental data on polymer structure, and it is therefore left for future studies.

It is worth noting that the proposed polymerization scheme (Fig. 9) implicitly supports the classical “loop-sheet” model for serpin polymerization [9]. Indeed, the occurrence of a fragmentation process, including the release of active monomers, adequately fits with a model, which does not foresee an extensive unfolding of proteins assembled into polymers, as (*e.g.*) in the “loop-sheet” model.

### Conclusive remarks

The polymerization of human neuroserpin was studied at different temperatures and concentrations. Our experiments show that linear polymeric chains are formed in a wide range of temperatures above the physiological one, with no other secondary mechanism (Figs. 2, 3, 4).

At the early stage of kinetics, the polymerization is driven by the process of activation of a monomeric intermediate conformer, prone to aggregation, and by the process of NS dimerization. The occurrence of these two processes had been already observed in a previous work [15], and it is here confirmed and characterized as the onset of the overall aggregation process. Then polymerization proceeds both by monomer addition to polymers and by polymer-polymer association. The latter process was revealed by the experiments here presented and was found to be diffusion-limited, at least in the late stage.

The most interesting observation emerging from our data is that NS polymerization is tempered by the occurrence of a fragmentation mechanism that likely also involves monomer release from polymers. Such a process is unique in the panorama of protein aggregation, where the most typical effect of fragmentation is an enhancement of the growth of aggregates. Moreover, the modulation of aggregate mass suggests a synchronization of the processes involved, in analogy with the observations on microtubule formation. A possible explanation of the observed modulation in the growth of neuroserpin polymers was found by the occurrence of latentization, that is the formation of inactive monomers, and the existence of relatively small fragile polymers.

The interplay among different process can thus provide a rationale for our experimental observation. We believe that some ingredient is still missing from the proposed model in order to expand the context of classical aggregation scheme. Further studies on the structure of neuroserpin polymers and latent monomers will help obtaining a quantitative explanation of the well-tempered aggregation of human neuroserpin.

## Materials and Methods

### Sample preparation

Recombinant NS was expressed and purified according to a previously published protocol [23]. The lack of cleaved NS was checked by gel electrophoresis (data not shown). A stock solution of a few mg/ml was prepared in 50 mM KCl, 10 mM HCl-Tris buffer pH 7.4, and stored at −80°C. The stock solution was diluted to the final concentration, aliquoted and kept at 4°C for a few days before experiments. Each aliquot was accurately filtered through Anatop 20 nm syringe-filters to remove large or small aggregates. The lack of aggregates was carefully checked by dynamic LS [39]. Protein concentration was measured by UV absorption at 280 nm (extinction coefficient: 0.803 cm^−1^ mg^−1^ ml^−1^). All chemicals were of reagent grade.

### Large Angle LS

Time-resolved dynamic LS experiments were performed at different temperatures. Samples were placed in a thermostated cell compartment of a Brookhaven Instruments BI200-SM goniometer, equipped with a solid-state laser tuned at *λ_0_* = 532 nm. The temperature was controlled within 0.05°C with a thermostated recirculating bath. Scattered light intensity and its time autocorrelation function *g_2_(t)* were measured simultaneously by using a Brookhaven BI-9000 correlator. Correlation functions *g_2_(t)* were analyzed by using a constrained regularization method in order to determine the distribution *P(R)* of the hydrodynamic radii *R*: *g_2_(t) = 1+|P(R) exp{−D(R)q^2^t} dR|^2^*. In the last expression, *q = 4πñλ_0_^−1^sin(ϑ/2)* is the scattering vector, with *ϑ* being the scattering angle and *ñ* the medium refractive index, and *D(R)* is the diffusion coefficient, related to R by the Stokes-Einstein relation: *D(R) = (k_B_T)/(6πηR)*, where *k_B_* is the Boltzmann constant, *T* is the temperature and *η* is the solvent viscosity [40]. Alternatively *g_2_(t)* were analyzed by fitting with a bimodal distribution function containing the monomer hydrodynamic radius R1 and the z-averaged polymer hydrodynamic radius *R_p_*, *g_2_(t) = 1+|A_1_exp{−D(R_1_)q_2_t}+A_p_exp{−D(R_p_)q_2_t}|^2^*, where *A_1_* and *A_p_* are the scattering amplitude related to the two contributions. From data analysis, one obtains the z-average hydrodynamic radius *R_h_*, which is defined as the harmonic average of the radii of the different species: *R_h_^−1^ = ∫P(R_h_^−1^) R_h_^−1^d R_h_^−1^*. Absolute values for scattered intensity (Rayleigh ratio) were obtained by normalization with respect to toluene, whose Rayleigh ratio at 532 nm was taken as 28·10^−6^ cm^−1^. From the Rayleigh ratio *R(q)*, at a given scattering vector *q*, one obtains the weight average mass *M_w_* of species in solution: *R(q) = HcM_w_P(q)*, where c is the total mass concentration, *P(q)* is the average form factor and *H = [2πñ dñ/dc λ_0_^−2^]^2^ N_A_^−1^* is a constant which depends upon the Avogadro number *N_A_*, the incident wavelength *λ_0_*, the refractive index of the medium *ñ* and the refractive index increment *dñ/dc*. The latter was assumed to be 0.18 cm^3^ g^−1^, a typical value for protein physiological solutions. The average form factor at 90° was computed by using the Guinier approximation, which holds for objects with a size small with respect to the inverse of the scattering vector: *P(q) = [1+1/3(qR_g_)^2^]^−1^*, where *R_g_* is the ratio of gyration. We estimated *R_g_* from the measured z-average hydrodynamic radius (*R_g_* = *R_h_*), which is a well-taken approximation for protein oligomers in solution [39]. As a matter of fact, for most of our experiments the form factor *P(q)* is essentially not relevant due to the moderate size of the objects in solution.

### Steady state PL

Steady state PL measurements were performed by incubating 4.5 µM NS samples in a quartz cuvette with a 3 mm optical path, in a thermostated cell holder of a Jasco FP-6500 spectrofluorimeter. The temperature was controlled within 0.05°C with a thermostated recirculating bath. The PL emission bands were measured with a 280 nm excitation wavelength, 3 nm excitation and emission bandwidth, 100 nm min^−1^ scan-speed, and 2 s response. The first momentum *M_1_* of the emission band was calculated as *M_1_ = hc [∫E L(E)dE]^−1^*, where *L(E)* is the normalized luminescence spectral profile at emission energy *E*, *h* is the Planck constant and *c* is the speed of light.

### Time lapsed SEC

Aggregate mass percentage was measured by SEC using a Phenomenex BioSep (SEC-S3000, 300×7.80 mm) column (Amersham) connected to a HPLC device (LC-2010 AT Prominence, Shimadzu, Kyoto, Japan), equipped with a UV-vis photodiode array detector and a 50 µL sample loop. Samples of 4.5 µM neuroserpin solution were incubated at 55°C for various time intervals in thermostated bath and directly transferred to the HPLC system. The samples were eluted at a flow of 0.8 mL min^−1^ in the sample buffer and absorption was measured at 280 nm. The percentage of monomers, dimers and larger polymers was estimated from the area of the related peaks with respect to the total area of eluate. The polymer, dimer and monomer peaks are taken within a range of elution volume of 5.0 to 8.1 ml, 8.1 to 8.6 ml and 8.6 ml to 12.0 ml, respectively.

## Supporting Information

Figure S1 **Transmission electron microscopy of neuroserpin polymers.** Polymers were formed by incubation of 0.5 mg ml^−1^ neuroserpin solutions overnight at 45°C (panel a) and for 2 hours at 85°C (panel b). A 10 µL aliquot was adsorbed on 200 mesh formvar/carbon grids for 5 minutes, washed with distilled water and negatively stained with 2% uranyl acetate. Imaging was obtained by a EFTEM Leo912 ab (Zeiss, Oberkochen, Germany) transmission electron microscope at 80 kV, equipped with Proscan 1K slowscan CCD.
(TIF)

Figure S2 **Neuroserpin polymerization at 85°C.** (a) Intensity autocorrelation functions at selected times during polymerization of a 1.5 mM neuroserpin solution at 85°C. (b) Kinetics of weight average mass M_w_ (black), z-average hydrodynamic radius R_h_ (red), characteristic time τ_slow_ (blue) and intensity I_slow_ (green) related to the slow decay. The polymer size increases in a few minutes from 85 nm to 500 nm. Afterwards, a second relaxation process appears in the intensity autocorrelation functions. This relaxation cannot be ascribed to a diffusional process and it was described by fitting with a compressed exponential function with exponent 2 and a characteristic time *t_slow_* = 200±25 ms. The correlation function were fit using the following expression: *g_2_(t) = 1+|A_p_*exp*{−D(R_p_)q^2^t}+A_slow_*exp*{−[t/t_slow_] ^2^}|^2^*. The amplitude and the characteristic time of such a slow process are reported in the next panel. The nature of this process is not clear. By considering analogous findings in gelling colloidal systems we may speculate that it is related to the constrained motion of large entangled or jammed polymer networks, as noted in the main text.
(PDF)

Table S1 **Kinetic scheme for neuroserpin polymerization.** The different molecular species and the different processes involved are listed, along with their rates.
(PDF)

Table S2 **Kinetic scheme for neuroserpin polymerization.** This set of equations describes the time evolution of monomeric and polymeric species. The last equation describes the time evolution of the experimentally accessible weight average mass M_w_, which is proportional to the second moment of the distribution of molecular species. The short notations defined in Table S1 are used, along with the notation O(t^3^) standing for terms of the order of t^3^ or higher. This general scheme was used to generate the curves of Fig. 8, by using the following expressions: *κ_A_ = 1*, *κ_D_ = 1*, *κ_R_ = κ_F_*, κ_L*_ = κ'_L*_ = 0, *a_Np_ = 1*, *a_Ip_ = 0*, *a_NI_ = 1*, *a_II_ = 0*, *b_Ip_ = 0*, *b_NN_ = b_II_ = b_NI_ = 0*, *b_Np_ = 1 for p<32*, *and b_Np_ = 0 for p>31*.
(PDF)
