# Supplementary figures and images for: The Tempered Polymerization of Human Neuroserpin
Source: PLoS One. 2012 Mar 6;7(3):e32444. doi: 10.1371/journal.pone.0032444 (PMC3295756; doi:10.1371/journal.pone.0032444)

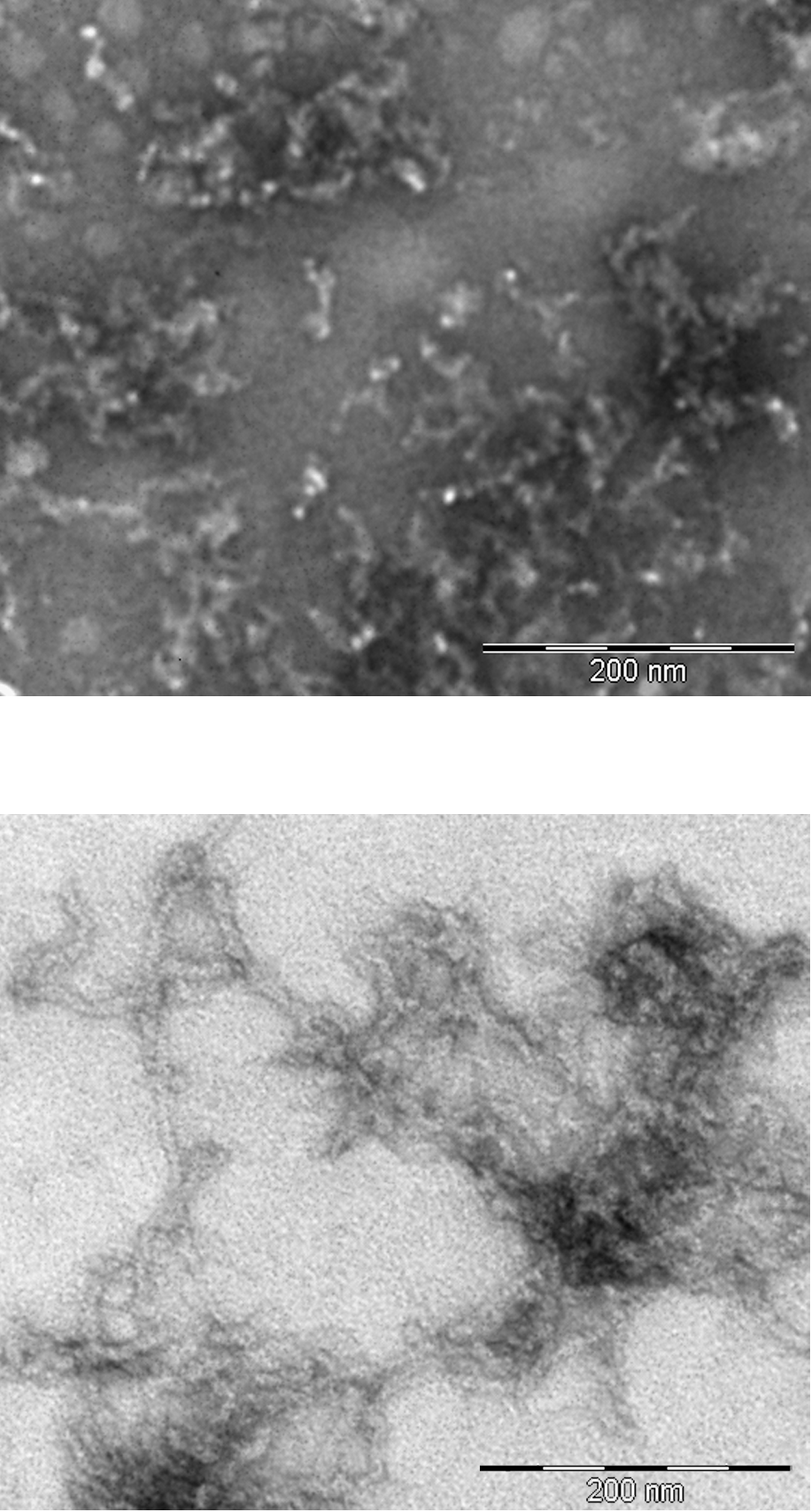

Supplement: Figure S1 — Transmission electron microscopy of neuroserpin polymers. Polymers were formed by incubation of 0.5 mg ml−1 neuroserpin solutions overnight at 45°C (panel a) and for 2 hours at 85°C (panel b). A 10 µL aliquot was adsorbed on 200 mesh formvar/carbon grids for 5 minutes, washed with distilled water and negatively stained with 2% uranyl acetate. Imaging was obtained by a EFTEM Leo912 ab (Zeiss, Oberkochen, Germany) transmission electron microscope at 80 kV, equipped with Proscan 1K slowscan CCD. (TIF) [file pone.0032444.s001.tif]

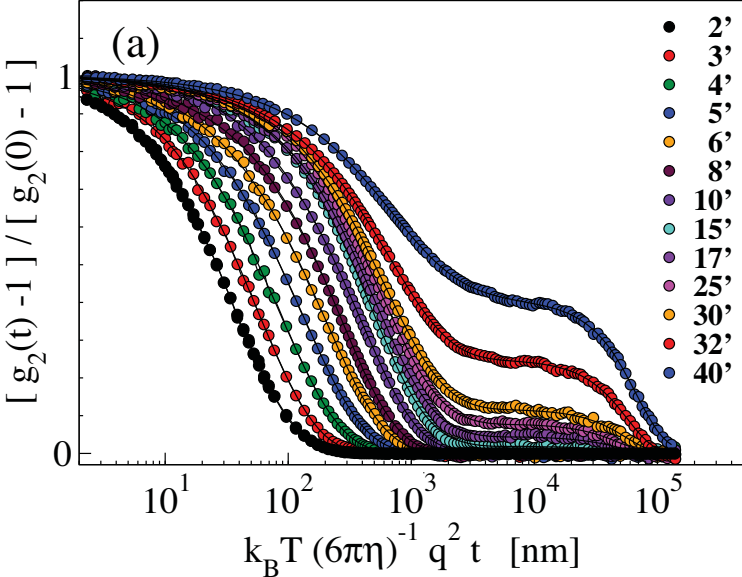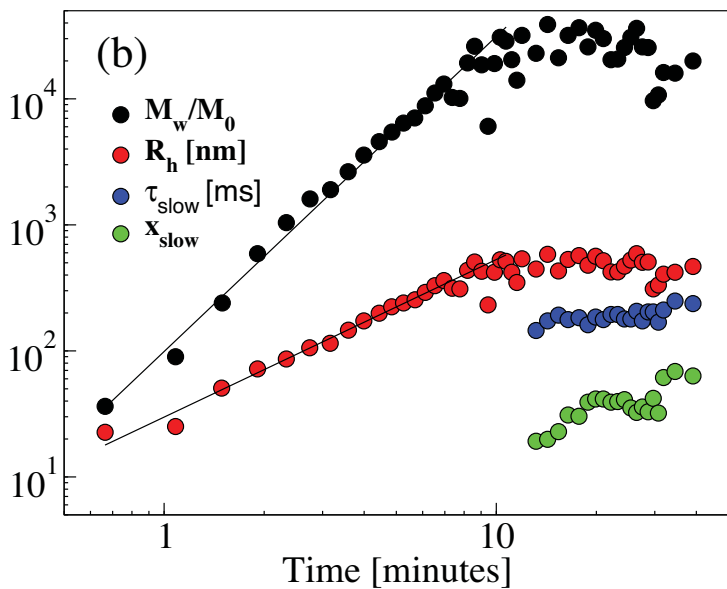

Supplement: Figure S2 — Neuroserpin polymerization at 85°C. (a) Intensity autocorrelation functions at selected times during polymerization of a 1.5 mM neuroserpin solution at 85°C. (b) Kinetics of weight average mass Mw (black), z-average hydrodynamic radius Rh (red), characteristic time τslow (blue) and intensity Islow (green) related to the slow decay. The polymer size increases in a few minutes from 85 nm to 500 nm. Afterwards, a second relaxation process appears in the intensity autocorrelation functions. This relaxation cannot be ascribed to a diffusional process and it was described by fitting with a compressed exponential function with exponent 2 and a characteristic time tslow = 200±25 ms. The correlation function were fit using the following expression: g2(t) = 1+|Apexp{−D(Rp)q2t}+Aslowexp{−[t/tslow] 2}|2. The amplitude and the characteristic time of such a slow process are reported in the next panel. The nature of this process is not clear. By considering analogous findings in gelling colloidal systems we may speculate that it is related to the constrained motion of large entangled or jammed polymer networks, as noted in the main text. (PDF) [file pone.0032444.s002.pdf]
