# Supplementary material for: The Tempered Polymerization of Human Neuroserpin
Source: PLoS One. 2012 Mar 6;7(3):e32444. doi: 10.1371/journal.pone.0032444 (PMC3295756; doi:10.1371/journal.pone.0032444)
Supplement: Table S1 — Kinetic scheme for neuroserpin polymerization. The different molecular species and the different processes involved are listed, along with their rates. (PDF) [file pone.0032444.s003.pdf]

Monomeric and polymeric species	Number concentrations
native functional neuroserpin	[N]
latent inactive neuroserpin	[L]
intermediate conformer, active for aggregation	[I]
polymer made of p monomers	[P] (1)

First order processes: molecular interconversions among monomeric states	Rates
activation of native monomers	$\kappa_A[N]$
latentization from native monomers	$\kappa_L[N]$ (2)
latentization from intermediates	$\kappa_L^*[I]$
inactivation of intermediates	$\kappa_A'[I]$
latent loss to native monomers	$\kappa_L'[L]$ (3)
latent loss to intermediates	$\kappa_L'^*[L]$ (3)

Second order processes: polymer formation	Rates (4)
dimerization from a native and an intermediate monomer	$a_{NI}\kappa_D c[N][I]$ (5)
dimerization from two intermediate monomers	$a_{II}\kappa_D c[I][I]$ (5)
native monomer addition to polymers of p monomers	$a_{Np}\kappa_M c[N][p]$ (6)
intermediate monomer addition to polymers of p monomers	$a_{Ip}\kappa_M c[I][p]$ (6)
association of two polymers of i and j monomers	$a_{ij}\kappa_P c[i][j]$

Other first order processes: depolymerization	Rates
native monomer release from polymers of p units	$b_{Np}\kappa_R[p]$ (7)
intermediate monomer release from polymers of p units	$b_{Ip}\kappa_R[p]$ (7)
fragmentation in two polymers of i and j units	$b_{ij}\kappa_F[i+j]$

(1) The total monomer number concentration is $[1] = [N] + [L] + [I]$
(2) This process has been ruled out by Chiou et al. 2009, Biophys. J. 97,2306.
(3) Latent loss can be reasonably neglected since latent conformation is in a deep free energy minimum. (4) c is the total mass concentration.
(5) Short notation for dimerization rates: $a_{11} = (a_{NI}[N] + a_{II}[I])\,[I][1]^{-2}\kappa_D\kappa_P^{-1}$.
(6) Short notation for monomer addition rates: $a_{1p} = (a_{Np}[N] + a_{Ip}[I])\,[1]^{-1}\kappa_M\kappa_P^{-1}$.
(7) Short notation for monomer release rates: $b_{1p} = (b_{Np} + b_{Ip})\,\kappa_R\kappa_F^{-1}$;
and for $p = 1$: $b_{1p} = (b_{NN} + b_{NI} + b_{II})\,\kappa_R\kappa_F^{-1}$.
