# Supplementary material for: The Tempered Polymerization of Human Neuroserpin
Source: PLoS One. 2012 Mar 6;7(3):e32444. doi: 10.1371/journal.pone.0032444 (PMC3295756; doi:10.1371/journal.pone.0032444)
Supplement: Table S2 — Kinetic scheme for neuroserpin polymerization. This set of equations describes the time evolution of monomeric and polymeric species. The last equation describes the time evolution of the experimentally accessible weight average mass Mw, which is proportional to the second moment of the distribution of molecular species. The short notations defined in Table S1 are used, along with the notation O(t3) standing for terms of the order of t3 or higher. This general scheme was used to generate the curves of Fig. 8, by using the following expressions: κA = 1, κD = 1, κR = κF, κL* = κ'L* = 0, aNp = 1, aIp = 0, aNI = 1, aII = 0, bIp = 0, bNN = bII = bNI = 0, bNp = 1 for p<32, and bNp = 0 for p>31. (PDF) [file pone.0032444.s004.pdf]

$$\frac{d}{dt}[N] = \underbrace{-\left(\ \kappa_{L*}[N] - \kappa'_{L*}[L]\ \right)}_{\text{Latentization}*} \underbrace{-\left(\ \kappa_A[N] - \kappa'_A[I]\ \right)}_{\text{Activation}} \underbrace{-\kappa_D a_{NI}[N][I]}_{\text{Dimerization}} \underbrace{+\kappa_R(2b_{NN} + b_{NI})[2]}_{\text{Dimer dissociation}}$$

$$\underbrace{-\kappa_M \sum_{p=2}^{\infty} a_{Np}[N][p]}_{\text{Monomer addition}} \underbrace{+\kappa_R \sum_{p=2}^{\infty} b_{Np}[p+1]}_{\text{Monomer release}}$$

$$\frac{d}{dt}[I] = \underbrace{-\left(\ \kappa_L[I] - \kappa'_L[L]\ \right)}_{\text{Latentization}} \underbrace{+\left(\ \kappa_A[N] - \kappa'_A[I]\ \right)}_{\text{Activation}} \underbrace{-\kappa_D\left(\ a_{NI}[N] + 2a_{II}[I]\right)[I]}_{\text{Dimerization}}$$

$$\underbrace{-\kappa_M \sum_{p=2}^{\infty} a_{Ip}[I][p]}_{\text{Monomer addition}} \underbrace{+\kappa_R \sum_{p=2}^{\infty} b_{Ip}[p+1]}_{\text{Monomer release}} \underbrace{+\kappa_R(2b_{II} + b_{NI})[2]}_{\text{Dimer dissociation}}$$

$$\frac{d}{dt}[L] = \underbrace{-\left(\ \kappa_L[I] - \kappa'_L[L]\ \right)}_{\text{Latentization}} \underbrace{-\left(\ \kappa_{L*}[N] - \kappa'_{L*}[L]\ \right)}_{\text{Latentization}*}$$

$$\underset{\{p>1\}}{\frac{d}{dt}[p]} = \underbrace{+\kappa_D\left(\ a_{NI}[N] + a_{II}[I]\ \right)[I]\ \delta_{p2}}_{\text{Dimerization}} \underbrace{+\kappa_R\left(\ b_{NI} + b_{NN} + b_{II}\ \right)[2]\ \delta_{p2}}_{\text{Dimer dissociation}}$$

$$\underbrace{+\kappa_M\left(\ a_{Np-1}[N] + a_{Ip-1}[I]\ \right)[p-1]\ (1-\delta_{p2}) - \kappa_M\left(\ a_{Np}[N] + a_{Ip}[I]\ \right)[p]}_{\text{Monomer addition}}$$

$$\underbrace{-\kappa_R\left(\ b_{Np-1} + b_{Ip-1}\ \right)[p]\ (1-\delta_{p2}) + \kappa_R\left(\ b_{Np} + b_{Ip}\ \right)[p+1]}_{\text{Monomer release}}$$

$$\underbrace{+\kappa_P \frac{1}{2} \sum_{j=2}^{p-2} a_{j,p-j}[j][p+j] - \kappa_P \sum_{j=2}^{\infty} a_{jp}[j][p]}_{\text{Polymer}-\text{PolymerAssociation}} \underbrace{-\kappa_F \frac{1}{2} \sum_{j=2}^{p-2} a_{j,p-j}[p] + \kappa_F \sum_{j=2}^{\infty} a_{jp}[p+j]}_{\text{Fragmentation}}$$

$$\frac{d}{dt}M_w = \frac{M_1^2}{c} \sum_{ij=1}^{\infty} ij\left\{\ a_{ij}c\kappa_P[i][j] - b_{ij}\kappa_F[i+j]\ \right\} = 1 + a_{NI}c\kappa_A\kappa_D t^2 + O\left(t^3\right)$$
